# 2,5-Bis(4-meth­oxy­phen­yl)-1,3,4-oxadiazole

**DOI:** 10.1107/S1600536810044405

**Published:** 2010-11-06

**Authors:** Hoong-Kun Fun, Jia Hao Goh, Nithinchandra, B. Kalluraya

**Affiliations:** a X-ray Crystallography Unit, School of Physics, Universiti Sains Malaysia, 11800 USM, Penang, Malaysia; b Department of Studies in Chemistry, Mangalore University, Mangalagangotri, Mangalore 574 199, India

## Abstract

In the title compound, C_16_H_14_N_2_O_3_, the essentially planar 1,3,4-oxadiazole ring [maximum deviation = 0.0021 (11) Å] is inclined at dihedral angles of 8.06 (6) and 11.21 (6)° with respect to the two benzene rings; the dihedral angle between the latter rings is 11.66 (5)°. In the crystal, short inter­molecular C⋯O inter­actions [2.9968 (15) Å] connect adjacent mol­ecules into chains propagating in [203]. The crystal structure is further stabilized by weak inter­molecular C—H⋯π inter­actions.

## Related literature

For general background to and applications of the title compound, see: Andersen *et al.* (1994 ▶); Clitherow *et al.* (1996 ▶); Hegde *et al.* (2008 ▶); Rai *et al.* (2008 ▶); Showell *et al.* (1991 ▶). For closely related 2,5-diphenyl-1,3,4-oxadiazole structures, see: Reck *et al.* (2003*a* ▶,*b* ▶); Franco *et al.* (2003 ▶). For the stability of the temperature controller used in the data collection, see: Cosier & Glazer (1986 ▶).
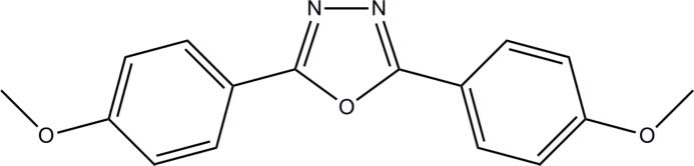


## Experimental

### 

#### Crystal data

C_16_H_14_N_2_O_3_
*M* *_r_* = 282.29
Monoclinic, 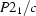

*a* = 10.7525 (2) Å
*b* = 11.8973 (2) Å
*c* = 11.6340 (2) Å
β = 115.434 (1)°
*V* = 1344.04 (4) Å^3^
*Z* = 4
Mo *K*α radiation
μ = 0.10 mm^−1^
*T* = 100 K
0.53 × 0.49 × 0.09 mm

#### Data collection

Bruker SMART APEXII CCD diffractometer
Absorption correction: multi-scan (*SADABS*; Bruker, 2009 ▶) *T*_min_ = 0.950, *T*_max_ = 0.992
17061 measured reflections
4744 independent reflections
3744 reflections with *I* > 2σ(*I*)
*R*_int_ = 0.027

#### Refinement

*R*[*F* ^2^ > 2σ(*F* ^2^)] = 0.047
*wR*(*F* ^2^) = 0.137
*S* = 1.03
4744 reflections
246 parameters
All H-atom parameters refined
Δρ_max_ = 0.38 e Å^−3^
Δρ_min_ = −0.30 e Å^−3^

### 

Data collection: *APEX2* (Bruker, 2009 ▶); cell refinement: *SAINT* (Bruker, 2009 ▶); data reduction: *SAINT*; program(s) used to solve structure: *SHELXTL* (Sheldrick, 2008 ▶); program(s) used to refine structure: *SHELXTL*; molecular graphics: *SHELXTL*; software used to prepare material for publication: *SHELXTL* and *PLATON* (Spek, 2009 ▶).

## Supplementary Material

Crystal structure: contains datablocks global, I. DOI: 10.1107/S1600536810044405/hb5714sup1.cif

Structure factors: contains datablocks I. DOI: 10.1107/S1600536810044405/hb5714Isup2.hkl

Additional supplementary materials:  crystallographic information; 3D view; checkCIF report

## Figures and Tables

**Table 1:** Hydrogen-bond geometry (Å, °) *Cg*1 and *Cg*2 are the centroids of C9–C14 and C1–C6 benzene rings, respectively.

*D*—H⋯*A*	*D*—H	H⋯*A*	*D*⋯*A*	*D*—H⋯*A*
C15—H15*A*⋯*Cg*1^i^	1.031 (14)	2.563 (16)	3.4903 (14)	149.4 (10)
C15—H15*B*⋯*Cg*2^ii^	0.992 (14)	2.994 (16)	3.8804 (14)	149.4 (11)

Symmetry codes: (i) 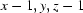; (ii) 
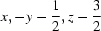
.

**Fig. 1.**
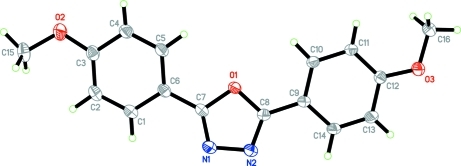
The asymmetric unit of the title compound, showing 50 % probability displacement ellipsoids for non-H atoms.

**Fig. 2.**
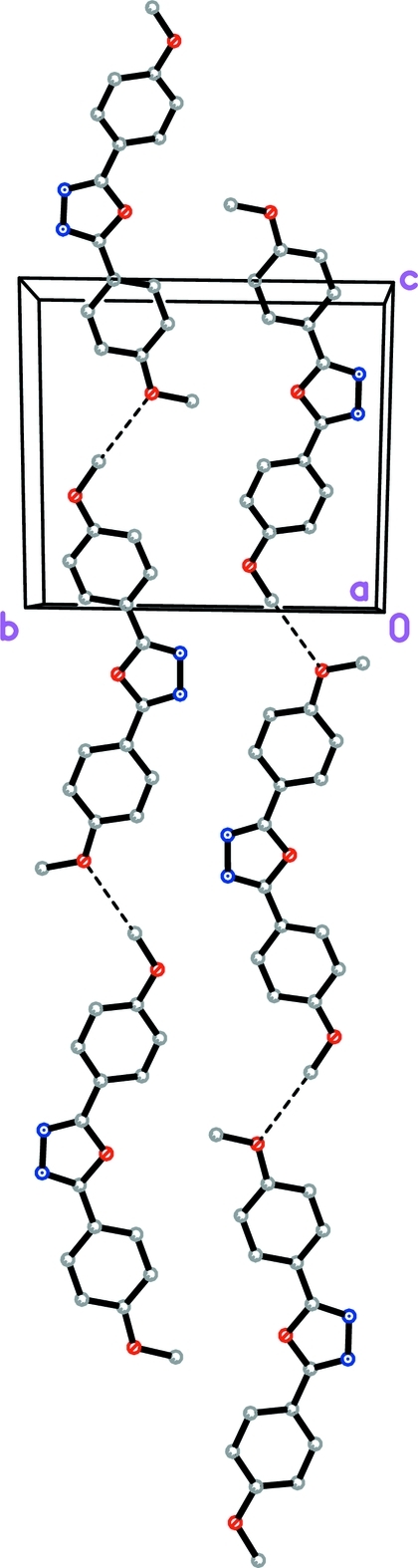
The crystal structure of the title compound, viewed along the a axis, showing molecules being interconnected into one-dimensional chains. H atoms have been omitted for clarity and intermolecular short interactions are shown as dashed lines.

## supplementary crystallographic information

### Comment

Heterocyclic compounds are becoming increasingly important in recent years due to their pharmacological activities (Rai *et al.*, 2008). Nitrogen- and oxygen-containing five/six-membered heterocyclic compounds are of enormous significance in the field of medicinal chemistry (Hegde *et al.*, 2008). Oxadiazoles play a very vital role in the preparation of various biologically active drugs with anti-inflammatory (Andersen *et al.*, 1994) and anti-cancer (Showell *et al.*, 1991) activities. The results of biological studies showed that oxadiazole derivatives also possess significant anti-inflammatory, analgesic and minimum ulcerogenic and lipid per-oxidation (Clitherow *et al.*, 1996) properties.

In the title oxadiazole compound (Fig. 1), the 1,3,4-oxadiazole ring (C7/C8/N1/N2/O1) is essentially planar, with a maximum deviation of -0.0021 (11) at atom N1. The C1-C6 and C9-C14 phenyl rings are inclined at dihedral angles of 11.21 (6) and 8.06 (6)°, respectively, with the 1,3,4-oxadiazole ring. The geometric parameters agree well with those observed in closely related 2,5-diphenyl-1,3,4-oxadiazole structures (Reck *et al.*, 2003*a*,*b*; Franco *et al.*, 2003).

In the crystal, no significant intermolecular hydrogen bond is observed. The interesting feature of the crystal structure is the intermolecular short C15···O3 interactions [2.9968 (15) Å, symmetry code: x+1, -y+1/2, z+3/2], which is significantly shorter than the sum of the Van der Waals radii of the relevant atoms (3.22 Å), interconnecting adjacent molecules into one-dimensional chains propagating along the [203] direction (Fig. 2). Further stabilization of the crystal structure is provided by weak intermolecular C15—H15A···*Cg*1 and C15—H15B···*Cg*2 interactions (Table 1) where *Cg*1 and *Cg*2 are the centroids of C9-C14 and C1-C6 phenyl rings, respectively.

### Experimental

An equimolar mixture of 4-methoxybenzoic acid (0.01 mol) and 4-methoxybenzhydrazide was dissolved in POCl_3_ (25–30 ml) by gentle warming. The solution is refluxed for about 12 h. Excess of POCl_3_ is distilled off, and then the reaction mixture is poured into crushed ice. The separated solid was filtered, washed with water and dried. It was then recrystallized from ethanol.  Yellow blocks of (I) were obtained from ethanol by slow evaporation.

### Refinement

All H atoms were located from difference Fourier map and allowed to refine freely with range of C—H = 0.916 (14) – 1.031 (14) Å.

### Crystal data

**Table:**

C_16_H_14_N_2_O_3_	*F*(000) = 592
*M**_r_* = 282.29	*D*_x_ = 1.395 Mg m^−^^3^
Monoclinic, *P*2_1_/*c*	Mo *K*α radiation, λ = 0.71073 Å
Hall symbol: -P 2ybc	Cell parameters from 5014 reflections
*a* = 10.7525 (2) Å	θ = 2.6–32.2°
*b* = 11.8973 (2) Å	µ = 0.10 mm^−^^1^
*c* = 11.6340 (2) Å	*T* = 100 K
β = 115.434 (1)°	Block, yellow
*V* = 1344.04 (4) Å^3^	0.53 × 0.49 × 0.09 mm
*Z* = 4	

### Data collection

**Table:**

Bruker SMART APEXII CCD diffractometer	4744 independent reflections
Radiation source: fine-focus sealed tube	3744 reflections with *I* > 2σ(*I*)
graphite	*R*_int_ = 0.027
φ and ω scans	θ_max_ = 32.3°, θ_min_ = 2.6°
Absorption correction: multi-scan (*SADABS*; Bruker, 2009)	*h* = −16→15
*T*_min_ = 0.950, *T*_max_ = 0.992	*k* = −17→17
17061 measured reflections	*l* = −14→17

### Refinement

**Table:**

Refinement on *F*^2^	Primary atom site location: structure-invariant direct methods
Least-squares matrix: full	Secondary atom site location: difference Fourier map
*R*[*F*^2^ > 2σ(*F*^2^)] = 0.047	Hydrogen site location: inferred from neighbouring sites
*wR*(*F*^2^) = 0.137	All H-atom parameters refined
*S* = 1.03	*w* = 1/[σ^2^(*F*_o_^2^) + (0.079*P*)^2^ + 0.1649*P*] where *P* = (*F*_o_^2^ + 2*F*_c_^2^)/3
4744 reflections	(Δ/σ)_max_ = 0.001
246 parameters	Δρ_max_ = 0.38 e Å^−^^3^
0 restraints	Δρ_min_ = −0.30 e Å^−^^3^

### Special details

**Table:**

Experimental. The crystal was placed in the cold stream of an Oxford Cryosystems Cobra open-flow nitrogen cryostat (Cosier & Glazer, 1986) operating at 100.0 (1)K.
Geometry. All esds (except the esd in the dihedral angle between two l.s. planes) are estimated using the full covariance matrix. The cell esds are taken into account individually in the estimation of esds in distances, angles and torsion angles; correlations between esds in cell parameters are only used when they are defined by crystal symmetry. An approximate (isotropic) treatment of cell esds is used for estimating esds involving l.s. planes.
Refinement. Refinement of F^2^ against ALL reflections. The weighted R-factor wR and goodness of fit S are based on F^2^, conventional R-factors R are based on F, with F set to zero for negative F^2^. The threshold expression of F^2^ > 2sigma(F^2^) is used only for calculating R-factors(gt) etc. and is not relevant to the choice of reflections for refinement. R-factors based on F^2^ are statistically about twice as large as those based on F, and R- factors based on ALL data will be even larger.

### Fractional atomic coordinates and isotropic or equivalent isotropic displacement parameters (Å^2^)

**Table:**

	*x*	*y*	*z*	*U*_iso_*/*U*_eq_	
O1	0.89032 (6)	0.25303 (6)	0.21170 (6)	0.01974 (15)	
O2	0.49848 (8)	0.12006 (7)	−0.35883 (8)	0.0333 (2)	
O3	1.26608 (8)	0.16634 (6)	0.80451 (7)	0.02542 (17)	
N1	0.84586 (9)	0.42831 (7)	0.14199 (9)	0.02542 (19)	
N2	0.93713 (9)	0.43051 (7)	0.27255 (8)	0.02513 (19)	
C1	0.64742 (10)	0.33907 (9)	−0.11316 (10)	0.0243 (2)	
C2	0.56651 (10)	0.29145 (9)	−0.23102 (10)	0.0257 (2)	
C3	0.57391 (10)	0.17639 (9)	−0.24904 (10)	0.0247 (2)	
C4	0.66395 (10)	0.10958 (9)	−0.14829 (10)	0.0264 (2)	
C5	0.74483 (10)	0.15763 (8)	−0.03171 (10)	0.0234 (2)	
C6	0.73715 (9)	0.27300 (8)	−0.01231 (9)	0.02039 (18)	
C7	0.82192 (9)	0.32333 (8)	0.11073 (9)	0.02031 (18)	
C8	0.95953 (9)	0.32608 (7)	0.30883 (9)	0.01932 (18)	
C9	1.04150 (9)	0.28123 (7)	0.43534 (9)	0.01863 (17)	
C10	1.06169 (9)	0.16598 (8)	0.45607 (9)	0.01989 (18)	
C11	1.13636 (9)	0.12351 (8)	0.57833 (9)	0.02060 (18)	
C12	1.19097 (9)	0.19768 (8)	0.68094 (9)	0.01974 (18)	
C13	1.17027 (10)	0.31372 (8)	0.66105 (10)	0.0232 (2)	
C14	1.09745 (10)	0.35469 (8)	0.53959 (10)	0.02276 (19)	
C15	0.42071 (11)	0.18563 (12)	−0.47047 (11)	0.0320 (2)	
C16	1.29532 (13)	0.05018 (10)	0.82938 (11)	0.0312 (2)	
H1A	0.6395 (13)	0.4157 (12)	−0.1004 (13)	0.031 (3)*	
H2A	0.5040 (16)	0.3408 (13)	−0.2994 (15)	0.044 (4)*	
H4A	0.6699 (14)	0.0323 (13)	−0.1637 (14)	0.034 (4)*	
H5A	0.8051 (14)	0.1104 (12)	0.0334 (13)	0.033 (4)*	
H10A	1.0288 (13)	0.1131 (11)	0.3879 (13)	0.028 (3)*	
H11A	1.1508 (14)	0.0475 (12)	0.5874 (14)	0.033 (3)*	
H13A	1.2011 (15)	0.3619 (13)	0.7306 (14)	0.039 (4)*	
H14A	1.0822 (13)	0.4342 (12)	0.5266 (13)	0.031 (3)*	
H15A	0.3446 (14)	0.2322 (11)	−0.4612 (13)	0.031 (3)*	
H15B	0.4821 (14)	0.2357 (12)	−0.4910 (14)	0.030 (3)*	
H15C	0.3802 (16)	0.1299 (14)	−0.5399 (15)	0.043 (4)*	
H16A	1.2087 (16)	0.0060 (13)	0.8025 (15)	0.044 (4)*	
H16B	1.3518 (15)	0.0229 (13)	0.7875 (14)	0.040 (4)*	
H16C	1.3490 (16)	0.0457 (14)	0.9238 (16)	0.046 (4)*	

### Atomic displacement parameters (Å^2^)

**Table:**

	*U*^11^	*U*^22^	*U*^33^	*U*^12^	*U*^13^	*U*^23^
O1	0.0201 (3)	0.0161 (3)	0.0210 (3)	0.0006 (2)	0.0069 (2)	0.0022 (2)
O2	0.0298 (4)	0.0347 (4)	0.0238 (4)	−0.0009 (3)	0.0003 (3)	0.0011 (3)
O3	0.0325 (4)	0.0234 (4)	0.0183 (3)	0.0021 (3)	0.0089 (3)	0.0011 (3)
N1	0.0316 (4)	0.0193 (4)	0.0244 (4)	0.0024 (3)	0.0112 (3)	0.0035 (3)
N2	0.0324 (4)	0.0184 (4)	0.0236 (4)	0.0022 (3)	0.0111 (3)	0.0020 (3)
C1	0.0247 (4)	0.0219 (5)	0.0262 (5)	0.0025 (3)	0.0108 (4)	0.0062 (4)
C2	0.0220 (4)	0.0283 (5)	0.0246 (5)	0.0024 (3)	0.0080 (4)	0.0086 (4)
C3	0.0197 (4)	0.0282 (5)	0.0224 (4)	−0.0015 (3)	0.0054 (3)	0.0031 (4)
C4	0.0243 (4)	0.0222 (5)	0.0265 (5)	0.0002 (3)	0.0050 (4)	0.0023 (4)
C5	0.0210 (4)	0.0217 (4)	0.0237 (4)	0.0009 (3)	0.0058 (3)	0.0050 (3)
C6	0.0183 (4)	0.0213 (4)	0.0219 (4)	0.0005 (3)	0.0090 (3)	0.0045 (3)
C7	0.0204 (4)	0.0192 (4)	0.0223 (4)	0.0031 (3)	0.0101 (3)	0.0054 (3)
C8	0.0210 (4)	0.0165 (4)	0.0217 (4)	0.0007 (3)	0.0103 (3)	−0.0001 (3)
C9	0.0192 (4)	0.0161 (4)	0.0213 (4)	0.0001 (3)	0.0092 (3)	−0.0003 (3)
C10	0.0201 (4)	0.0161 (4)	0.0220 (4)	−0.0010 (3)	0.0076 (3)	−0.0020 (3)
C11	0.0222 (4)	0.0156 (4)	0.0228 (4)	−0.0009 (3)	0.0086 (3)	−0.0005 (3)
C12	0.0212 (4)	0.0206 (4)	0.0185 (4)	0.0006 (3)	0.0096 (3)	0.0004 (3)
C13	0.0284 (4)	0.0195 (4)	0.0223 (4)	0.0010 (3)	0.0113 (4)	−0.0039 (3)
C14	0.0275 (4)	0.0163 (4)	0.0246 (4)	0.0017 (3)	0.0112 (4)	−0.0015 (3)
C15	0.0237 (4)	0.0470 (7)	0.0213 (5)	0.0004 (4)	0.0059 (4)	0.0067 (4)
C16	0.0390 (6)	0.0239 (5)	0.0242 (5)	0.0018 (4)	0.0074 (4)	0.0053 (4)

### Geometric parameters (Å, °)

**Table:**

O1—C8	1.3652 (11)	C6—C7	1.4552 (13)
O1—C7	1.3704 (11)	C8—C9	1.4537 (13)
O2—C3	1.3597 (13)	C9—C10	1.3930 (12)
O2—C15	1.4359 (13)	C9—C14	1.4036 (13)
O3—C12	1.3641 (11)	C10—C11	1.3937 (13)
O3—C16	1.4193 (13)	C10—H10A	0.954 (14)
N1—C7	1.2954 (13)	C11—C12	1.3952 (13)
N1—N2	1.4110 (12)	C11—H11A	0.916 (14)
N2—C8	1.3010 (12)	C12—C13	1.4017 (14)
C1—C2	1.3905 (15)	C13—C14	1.3774 (14)
C1—C6	1.3975 (13)	C13—H13A	0.929 (16)
C1—H1A	0.934 (14)	C14—H14A	0.960 (14)
C2—C3	1.3923 (15)	C15—H15A	1.031 (14)
C2—H2A	0.985 (16)	C15—H15B	0.992 (14)
C3—C4	1.4029 (14)	C15—H15C	0.990 (16)
C4—C5	1.3819 (14)	C16—H16A	0.996 (16)
C4—H4A	0.944 (15)	C16—H16B	0.982 (16)
C5—C6	1.3992 (14)	C16—H16C	0.999 (17)
C5—H5A	0.943 (14)		
			
C8—O1—C7	102.83 (7)	C10—C9—C8	121.21 (8)
C3—O2—C15	117.56 (9)	C14—C9—C8	119.66 (8)
C12—O3—C16	117.39 (8)	C9—C10—C11	120.82 (8)
C7—N1—N2	106.41 (8)	C9—C10—H10A	122.1 (8)
C8—N2—N1	106.13 (8)	C11—C10—H10A	117.1 (8)
C2—C1—C6	120.85 (9)	C10—C11—C12	119.35 (9)
C2—C1—H1A	119.5 (9)	C10—C11—H11A	118.0 (9)
C6—C1—H1A	119.6 (9)	C12—C11—H11A	122.5 (9)
C1—C2—C3	119.80 (9)	O3—C12—C11	124.75 (9)
C1—C2—H2A	118.4 (9)	O3—C12—C13	115.05 (8)
C3—C2—H2A	121.8 (9)	C11—C12—C13	120.20 (9)
O2—C3—C2	125.20 (9)	C14—C13—C12	119.87 (9)
O2—C3—C4	115.14 (9)	C14—C13—H13A	120.6 (10)
C2—C3—C4	119.66 (9)	C12—C13—H13A	119.5 (10)
C5—C4—C3	120.21 (10)	C13—C14—C9	120.66 (9)
C5—C4—H4A	121.6 (9)	C13—C14—H14A	119.5 (8)
C3—C4—H4A	118.2 (9)	C9—C14—H14A	119.8 (8)
C4—C5—C6	120.55 (9)	O2—C15—H15A	112.1 (8)
C4—C5—H5A	117.9 (9)	O2—C15—H15B	110.8 (8)
C6—C5—H5A	121.6 (9)	H15A—C15—H15B	110.0 (11)
C1—C6—C5	118.92 (9)	O2—C15—H15C	104.8 (10)
C1—C6—C7	120.62 (9)	H15A—C15—H15C	110.8 (12)
C5—C6—C7	120.46 (8)	H15B—C15—H15C	108.1 (12)
N1—C7—O1	112.27 (9)	O3—C16—H16A	110.8 (9)
N1—C7—C6	129.63 (9)	O3—C16—H16B	110.5 (9)
O1—C7—C6	118.09 (8)	H16A—C16—H16B	111.3 (13)
N2—C8—O1	112.35 (8)	O3—C16—H16C	104.4 (10)
N2—C8—C9	128.77 (9)	H16A—C16—H16C	109.8 (13)
O1—C8—C9	118.84 (8)	H16B—C16—H16C	109.8 (13)
C10—C9—C14	119.09 (9)		
			
C7—N1—N2—C8	−0.41 (11)	N1—N2—C8—O1	0.33 (11)
C6—C1—C2—C3	0.44 (16)	N1—N2—C8—C9	−177.28 (9)
C15—O2—C3—C2	9.99 (16)	C7—O1—C8—N2	−0.13 (10)
C15—O2—C3—C4	−170.60 (10)	C7—O1—C8—C9	177.74 (8)
C1—C2—C3—O2	178.82 (10)	N2—C8—C9—C10	−175.90 (10)
C1—C2—C3—C4	−0.55 (16)	O1—C8—C9—C10	6.62 (13)
O2—C3—C4—C5	−179.38 (10)	N2—C8—C9—C14	6.42 (16)
C2—C3—C4—C5	0.05 (16)	O1—C8—C9—C14	−171.05 (8)
C3—C4—C5—C6	0.57 (16)	C14—C9—C10—C11	−0.04 (14)
C2—C1—C6—C5	0.18 (15)	C8—C9—C10—C11	−177.72 (9)
C2—C1—C6—C7	179.80 (9)	C9—C10—C11—C12	0.20 (14)
C4—C5—C6—C1	−0.69 (15)	C16—O3—C12—C11	2.71 (14)
C4—C5—C6—C7	179.69 (9)	C16—O3—C12—C13	−177.29 (9)
N2—N1—C7—O1	0.35 (11)	C10—C11—C12—O3	−179.73 (8)
N2—N1—C7—C6	−178.82 (9)	C10—C11—C12—C13	0.27 (14)
C8—O1—C7—N1	−0.15 (10)	O3—C12—C13—C14	179.08 (9)
C8—O1—C7—C6	179.12 (8)	C11—C12—C13—C14	−0.92 (15)
C1—C6—C7—N1	−11.61 (16)	C12—C13—C14—C9	1.10 (15)
C5—C6—C7—N1	168.01 (10)	C10—C9—C14—C13	−0.62 (14)
C1—C6—C7—O1	169.27 (9)	C8—C9—C14—C13	177.10 (9)
C5—C6—C7—O1	−11.12 (13)		

### Hydrogen-bond geometry (Å, °)

**Table:**

Cg1 and Cg2 are the centroids of C9–C14 and C1–C6 benzene rings, respectively.

**Table:**

*D*—H···*A*	*D*—H	H···*A*	*D*···*A*	*D*—H···*A*
C15—H15A···Cg1^i^	1.031 (14)	2.563 (16)	3.4903 (14)	149.4 (10)
C15—H15B···Cg2^ii^	0.992 (14)	2.994 (16)	3.8804 (14)	149.4 (11)

Symmetry codes: (i) *x*−1, *y*, *z*−1; (ii) *x*, −*y*−1/2, *z*−3/2.
